# Tryptophan Metabolism via the Kynurenine Pathway: Implications for Graft Optimization during Machine Perfusion

**DOI:** 10.3390/jcm9061864

**Published:** 2020-06-15

**Authors:** Anna Zhang, Cailah Carroll, Siavash Raigani, Negin Karimian, Viola Huang, Sonal Nagpal, Irene Beijert, Robert J. Porte, Martin Yarmush, Korkut Uygun, Heidi Yeh

**Affiliations:** 1 Center for Engineering in Medicine and Surgery, Massachusetts General Hospital, Harvard Medical School, Boston, MA 02114, USA; Anna.Zhang@tufts.edu (A.Z.); cailah.carroll@uconn.edu (C.C.); sraigani@mgh.harvard.edu (S.R.); n.karimian284@gmail.com (N.K.); vhuang450@gmail.com (V.H.); nsonal400@gmail.com (S.N.); ibie65552@gmail.com (I.B.); MYARMUSH@mgh.harvard.edu (M.Y.); KUYGUN@mgh.harvard.edu (K.U.); 2 Tufts University School of Medicine, Boston, MA 02111, USA; 3 Shriners Hospital for Children, Boston, MA 02114, USA; 4 Division of Transplant Surgery, Massachusetts General Hospital, Harvard Medical School, Boston, MA 02114, USA; 5 Division of Hepatobiliary Surgery and Liver Transplantation, University Medical Center Groningen, 9700 Groningen, The Netherlands; r.j.porte@umcg.nl; 6 Department of Biomedical Engineering, Rutgers University, Piscataway, NJ 08854, USA

**Keywords:** kynurenine, tryptophan, histamine, glutathione, machine perfusion, liver transplant, metabolomics, normothermic, subnormothermic, ex situ perfusion

## Abstract

Access to liver transplantation continues to be hindered by the severe organ shortage. Extended-criteria donor livers could be used to expand the donor pool but are prone to ischemia-reperfusion injury (IRI) and post-transplant graft dysfunction. Ex situ machine perfusion may be used as a platform to rehabilitate discarded or extended-criteria livers prior to transplantation, though there is a lack of data guiding the utilization of different perfusion modalities and therapeutics. Since amino acid derivatives involved in inflammatory and antioxidant pathways are critical in IRI, we analyzed differences in amino acid metabolism in seven discarded non-steatotic human livers during normothermic- (NMP) and subnormothermic-machine perfusion (SNMP) using data from untargeted metabolomic profiling. We found notable differences in tryptophan, histamine, and glutathione metabolism. Greater tryptophan metabolism via the kynurenine pathway during NMP was indicated by significantly higher kynurenine and kynurenate tissue concentrations compared to pre-perfusion levels. Livers undergoing SNMP demonstrated impaired glutathione synthesis indicated by depletion of reduced and oxidized glutathione tissue concentrations. Notably, ATP and energy charge ratios were greater in livers during SNMP compared to NMP. Given these findings, several targeted therapeutic interventions are proposed to mitigate IRI during liver machine perfusion and optimize marginal liver grafts during SNMP and NMP.

## 1. Introduction

Liver transplantation (LT) remains the only definitive cure for end-stage liver diseases. However, the number of patients waiting for LT continues to increase due to the shortage of organs while waitlist mortality remains high [1]. One solution to remedy the severe organ shortage is to improve the utilization of extended-criteria donor (ECD) livers. The current limitation to the widespread use of ECD livers is the increased severity of post-transplant ischemia-reperfusion injury (IRI), especially as a result of warm ischemia during donation after circulatory death (DCD). As a result, many ECD livers are discarded due to concerns for early allograft dysfunction or primary nonfunction. Machine perfusion (MP) devices have emerged as a promising platform for both viability assessment and delivery of novel therapeutics aimed at rehabilitating suboptimal grafts for transplantation [2].

Despite the advent of machine perfusion, the debate continues regarding the best practices. Currently, most transplant centers use either normothermic machine perfusion (NMP) at 37 °C [3] or hypothermic oxygenated perfusion (HOPE) at 2–10 °C [4,5]. Controlled oxygenated rewarming (COR) after static cold storage has also demonstrated improved graft function in animal and human trials [6,7,8]. Subnormothermic machine perfusion (SNMP) at 20–25 °C [4,9], however, remains largely experimental.

Many physicians envision a future where MP is used to improve the function of each donor liver to achieve maximal graft utilization and minimize discard rates [10,11], and one potential method to improve graft function is targeting metabolism. While numerous studies have demonstrated potential metabolic targets in the liver, renal, and intestinal IRI response [12,13,14,15,16], few studies have examined the metabolic changes that occur in the human liver during transplantation [12,17,18,19,20] and fewer still during ex situ machine perfusion [4,21]. This is a critical area of research since the machine perfusion platform is the only setting for liver-specific adjunct therapies prior to transplantation. To address this knowledge gap, we sought to assess the metabolomic profiles of discarded human livers without significant steatosis (<20% macrosteatosis) during SNMP and NMP. We hypothesized was that metabolic changes taking place in the liver driven by different perfusion modalities would identify potentially harmful and beneficial processes, which can be used to optimize future perfusion outcomes by mitigating IRI. These insights provide valuable knowledge that can be used to generate new hypotheses and design therapeutic interventions to salvage or rehabilitate discarded or ECD livers for transplantation.

## 2. Materials and Methods

### 2.1. Donor Livers

Seven human donor livers with <20% macrosteatosis that were declined for transplant were obtained through New England Donor Services (NEDS). Livers with evidence of cirrhosis, significant steatosis, or major trauma were excluded. Table 1 describes the reason each organ was declined for transplantation. The Massachusetts General Hospital Institutional Review Board (IRB) and NEDS approved this study (No. 2011P001496). No organs were procured from prisoners and no vulnerable populations were included in this study.

### 2.2. Procurement of Grafts

Procurement techniques based on donation after brain death (DBD) and donation after cardiac death (DCD) followed standard methods [9]. Donor livers were flushed in situ with cold University of Wisconsin (UW) preservation solution. Warm ischemic time (WIT) was defined as the period between extubation and in situ cold flush. Cold ischemic time (CIT) was defined as the period between in situ cold flush and initiation of machine perfusion. Livers arrived in the laboratory under static cold storage and underwent back table preparation according to previously described methods [22].

### 2.3. Machine Perfusion

Livers underwent three hours of either NMP or SNMP using the Liver Assist device (Organ Assist, Groningen, Netherlands). Previous studies have shown the liver reaches a steady-state within this time frame with regards to perfusion dynamics, biomarkers, and energy capacity [9,23]. Clinically, viability assessment is also performed within this time frame [24]. The temperatures of the NMP and SNMP circuits were maintained between 35–37 °C and 20–22 °C, respectively. Perfusate composition has been previously described [21] but is notably different between the groups given the addition of a hemoglobin-based oxygen carrier, HBOC-201 (HbO2 Therapeutics LLC, Souderton, PA, USA) in the NMP group. Detailed perfusate compositions are provided in the Supplementary Materials (Table S1). SNMP livers were perfused at a mean hepatic arterial pressure (HAP) of 30–60 mmHg and a portal venous pressure (PVP) of 3–7 mmHg. NMP livers were perfused at a HAP of 60–70 mmHg and a PVP of 6–8 mmHg. Detailed methods for sample collection have been previously described [21].

### 2.4. Energy Cofactor Analysis

Wedge liver biopsies collected hourly were frozen, pulverized in liquid nitrogen, and analyzed for metabolic cofactors. The targeted multiple reaction monitoring analysis was performed at the principle research institution on a Sciex TripleTOF 6600 Quadruple Time-of-Flight system. Metabolite extraction was conducted according to a previously described protocol [25]. Concentrations of hepatic adenosine mono-, di-, and tri- phosphate (AMP, ADP, and ATP) were quantified. Energy charge was calculated as: [ATP + ADP*0.5] / [ATP + ADP + AMP].

### 2.5. Untargeted Metabolomic Analysis

Liver biopsies were further analyzed by Metabolon, Inc. (Durham, NC, USA) for 1600 known compounds. Analyses were performed using ultrahigh performance liquid chromatography-tandem mass spectroscopy (UPLC-MS/MS). Detailed methods and statistical approaches have been previously described [21].

### 2.6. Statistical Analysis

Demographic and perfusion data are presented as the median ± interquartile range (IQR), unless otherwise specified, with statistical significance defined as *p* < 0.05. Wilcoxon’s rank-sum (Mann–Whitney U) test and Fischer’s exact test were used for continuous and categorical comparisons, respectively. A random intercept mixed model with a categorical effect of time was used to analyze repeated measures data. Stata 15.1 (StataCorp, College Station, TX, USA) was used to perform statistical analyses and Prism 8 (GraphPad, San Diego, CA, USA) was used to create graphics.

Statistical analysis for untargeted metabolomic profiling has been previously described [21]. Fold changes of metabolites are presented as the mean with statistical significance defined as *p* ≤ 0.05.

## 3. Results

### 3.1. Perfusion and Functional Parameters

**Figure 1:**
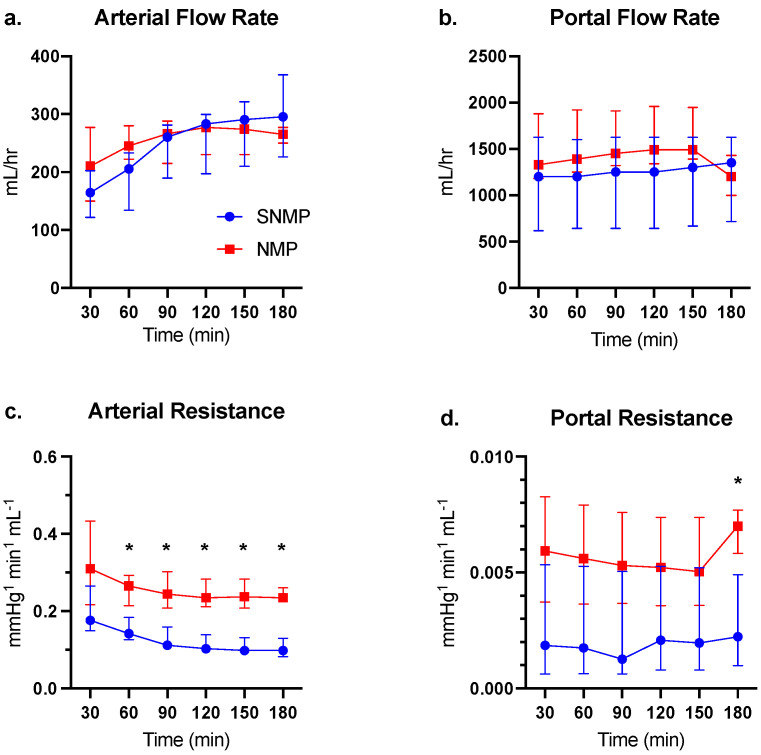
Hemodynamics of discarded human non-steatotic livers during SNMP vs. NMP. (**a**) Arterial and (**b**) portal flows were similar between groups during three hours of perfusion. Both NMP and SNMP livers demonstrate a steady increase in arterial flow. (**c**) Arterial resistance was significantly higher in the NMP group after the initiation of perfusion. (**d**) Portal resistance tended to be higher in the NMP group but only reached significance after 180 min of perfusion. * indicates *p* < 0.05 for comparisons using Wilcoxon’s rank-sum test. Abbreviations: SNMP, subnormothermic machine perfusion; NMP, normothermic machine perfusion.

Baseline characteristics of the NMP and SNMP livers are provided in Table 2. Arterial and portal flow rates were comparable between the two groups (Figure 1a,b). NMP livers demonstrated significantly higher arterial resistance after 60 min of perfusion compared to the SNMP group (Figure 1c). Portal resistance tended to be higher in the NMP group, but only reached significance after 180 min of perfusion (Figure 1d).

**Figure 2:**
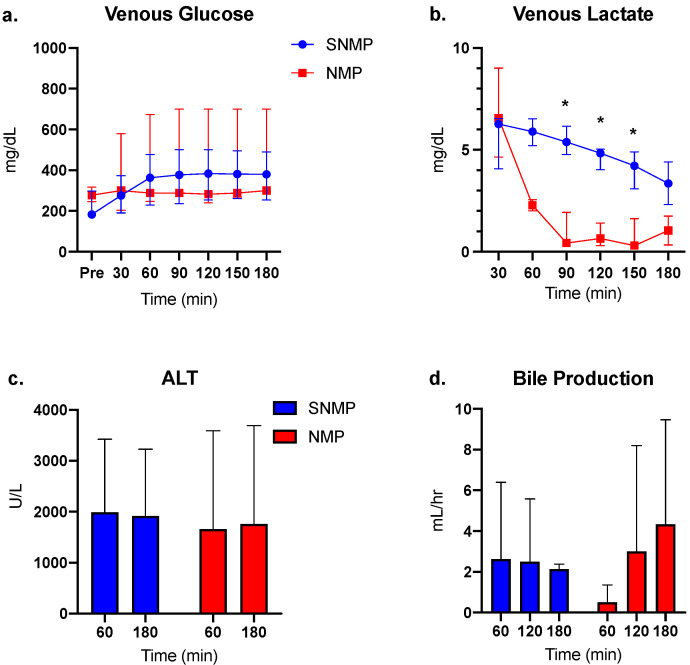
Biomarkers of liver function during SNMP vs. NMP. (**a**) Venous (perfusate) glucose levels were similar between the two groups throughout perfusion, though variance was greater in the NMP group. (**b**) Venous lactate was rapidly cleared during NMP compared to a slower decline during SNMP. (**c**) Alanine aminotransferase (ALT) levels were similar between groups at 60 and 180 min of perfusion. (**d**) Bile production increased at each hour of NMP but was statistically similar between groups. * indicates *p* < 0.05 for Wilcoxon’s rank-sum test. Abbreviations: SNMP, subnormothermic machine perfusion; NMP, normothermic machine perfusion.

Perfusate glucose levels were comparable between the two groups but varied widely in the NMP group (Figure 2a). Venous lactate levels were comparable between the groups at the initiation of perfusion but were cleared at a significantly faster rate in the NMP group, as expected (Figure 2b). ALT levels were comparable between the groups at 60 and 180 min of perfusion, though within-group variance was large (Figure 2c). Bile was produced in consistent volumes during each hour of SNMP compared to increasing volumes during NMP (Figure 2d).

### 3.2. Greater ATP Conservation and Energy Charge Ratios during SNMP

**Figure 3:**
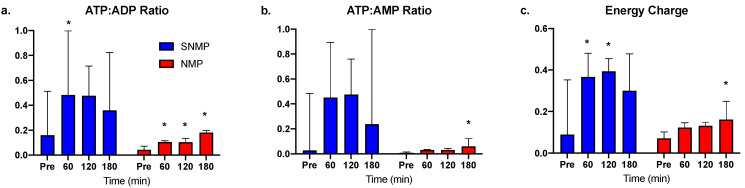
Energy cofactor changes during SNMP vs. NMP. (**a**) ATP:ADP, (**b**) ATP:AMP, and (**c**) energy charge ratios were quantitatively much higher in the SNMP group after initiation of perfusion, but with large variance. Livers undergoing NMP demonstrate a consistent increase in energy cofactor, though absolute values were lower compared to SNMP indicating the more active metabolic state at physiologic temperatures. * indicates *p* < 0.05 for the random intercept mixed model. Abbreviations: ATP, adenosine triphosphate; ADP, adenosine diphosphate; AMP, adenosine monophosphate; SNMP, subnormothermic machine perfusion; NMP, normothermic machine perfusion.

Compared to pre-perfusion levels, ATP:ADP ratios increased at each hour of SNMP but only reached significance at 60 min (Figure 3a). ATP:ADP ratios increased significantly at each hour of NMP, though absolute ratio values were smaller compared to the SNMP group. A similar trend was seen with ATP:AMP ratio (Figure 3b) and energy charge (Figure 3c).

### 3.3. Greater Tryptophan Metabolism in Livers during NMP

**Figure 4:**
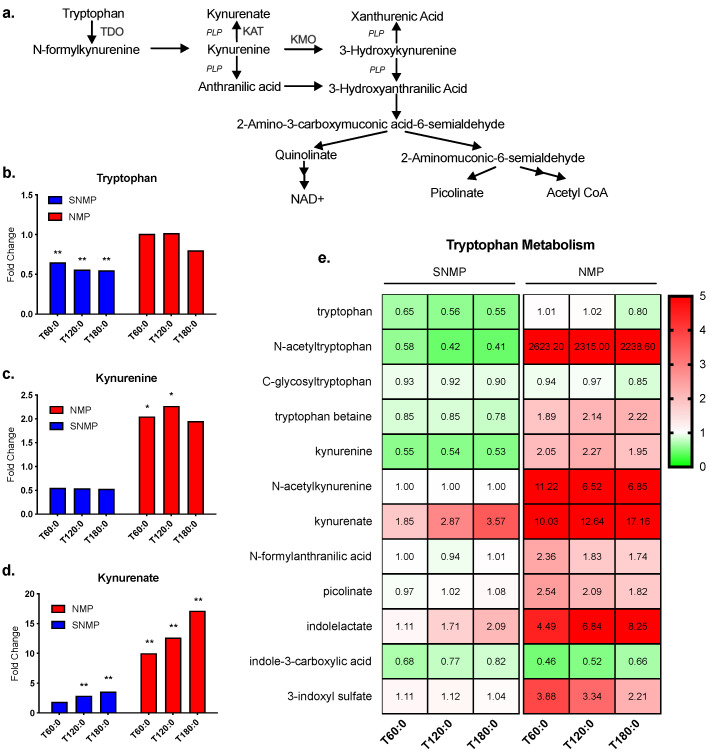
Tryptophan metabolism via the kynurenine pathway during SNMP vs. NMP. (**a**) Tryptophan metabolism via the kynurenine pathway. PLP is a necessary cofactor for several steps in the pathway. (**b**) SNMP livers demonstrate a significant decrease in liver tryptophan concentrations compared to NMP livers. However, NMP livers demonstrate a significant increase in tissue (**c**) kynurenine and (**d**) kynurenate compared to the SNMP group. (**e**) Heatmap of metabolites involved in tryptophan metabolites analyzed in the untargeted metabolomic analysis. ** indicates *p* ≤ 0.05 and * indicates 0.05 < *p* < 0.10. Abbreviations: TDO, tryptophan 2,3-dioxygenase; KAT, kynurenine aminotransferase; KMO, kynurenine 3-monooxygenase; PLP, pyridoxal 5-phosphate, NAD+, nicotinamide adenine nucleotide; SNMP, subnormothermic machine perfusion; NMP, normothermic machine perfusion; x-axis represents fold change at 60, 120, and 180 min compared to pre-perfusion concentrations.

The kynurenine pathway generates nicotinamide adenine dinucleotide (NAD+) from tryptophan, in addition to several other notable metabolites (Figure 4a) [26]. Tryptophan levels decreased significantly in SNMP livers (fold change range 0.55–0.65, *p* ≤ 0.05 for all) but showed no significant change in NMP livers (fold change range 0.80–1.02, *p* not significant) (Figure 4b). Kynurenine fold change ratios decreased non-significantly during SNMP but increased during NMP (fold change range 1.95–2.27, 0.05 < *p* < 0.10 at 60 and 120 min, *p* not significant at 180 min) (Figure 4c). Kynurenate fold change ratios increased significantly in SNMP livers (fold change range 1.85–3.57, *p* ≤ 0.05 at 120 and 180 min) and to an even larger degree in NMP livers (fold change range 10.03–17.16, *p* ≤ 0.05 for all) (Figure 4d). The fold change ratios of tryptophan metabolites largely increased during NMP compared to SNMP. The one exception to this pattern was indole-3-carboxylic acid, which decreased during NMP (Figure 4e). No significant changes were seen in NAD+ levels during NMP or SNMP (Supplementary Materials, Figure S1).

**Figure 5:**
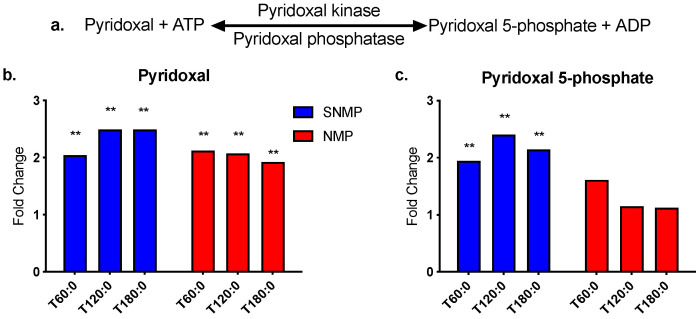
Vitamin B6 metabolism during SNMP vs. NMP. (**a**) Pyridoxal 5-phosphate (PLP) is generated from pyridoxal in a reversible reaction requiring ATP. SNMP livers demonstrate a significant increase in both (**b**) pyridoxal and (**c**) PLP levels during perfusion, whereas NMP livers only demonstrate a significant increase in pyridoxal. ** indicates *p* ≤ 0.05 and * indicates 0.05 < *p* < 0.10. Abbreviations: ATP, adenosine triphosphate; ADP, adenosine diphosphate; PLP, pyridoxal 5-phosphate; SNMP, subnormothermic machine perfusion; NMP, normothermic machine perfusion; x-axis represents fold change at 60, 120, and 180 min compared to pre-perfusion concentrations.

Biologically active vitamin B6 exists as six interconvertible compounds: pyridoxine, pyridoxine 5-phosphate, pyridoxamine, pyridoxamine 5-phosphate, pyridoxal, and pyridoxal 5-phosphate (PLP). PLP is a necessary coenzyme for several steps of tryptophan metabolism via the kynurenine pathway and is synthesized from pyridoxal at the expense of one ATP molecule (Figure 5a) [27]. Pyridoxal levels increased significantly in both temperature modalities, while PLP increased significantly only during SNMP (fold change range 1.94–2.4, *p* ≤ 0.05 for all) (Figure 5b,c).

### 3.4. Greater Histamine Reduction in Livers during SNMP

**Figure 6:**
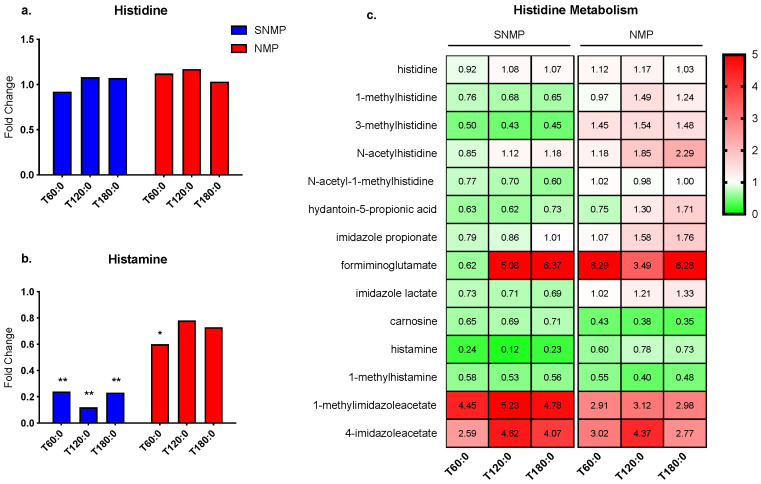
Histidine metabolism during SNMP vs. NMP. (**a**) Histidine concentrations are unchanged during SNMP and NMP, whereas (**b**) histamine tissue concentrations are significantly decreased during SNMP. (**c**) Heatmap showing concentration fold changes of histidine metabolites during SNMP and NMP. ** indicates *p* ≤ 0.05 and * indicates 0.05 < *p* < 0.10. Abbreviations: SNMP, subnormothermic machine perfusion; NMP, normothermic machine perfusion; x-axis represents fold change at 60, 120, and 180 min compared to pre-perfusion concentrations.

Histamine, formed from its amino acid precursor histidine, is a pro-inflammatory mediator involved in many diverse inflammatory and immune responses [28]. The liver is also an important location of excess histamine clearance [29]. No significant changes were seen in histidine in livers undergoing NMP or SNMP (Figure 6a). However, histamine levels decreased in both groups, though to a much larger degree during SNMP (fold change range 0.12–0.24, *p* ≤ 0.05 for all) (Figure 6b). Similarly, most histidine metabolites demonstrated an overall decrease during SNMP compared to NMP (Figure 6c).

### 3.5. Decreased Antioxidant Capacity in Livers during SNMP

**Figure 7:**
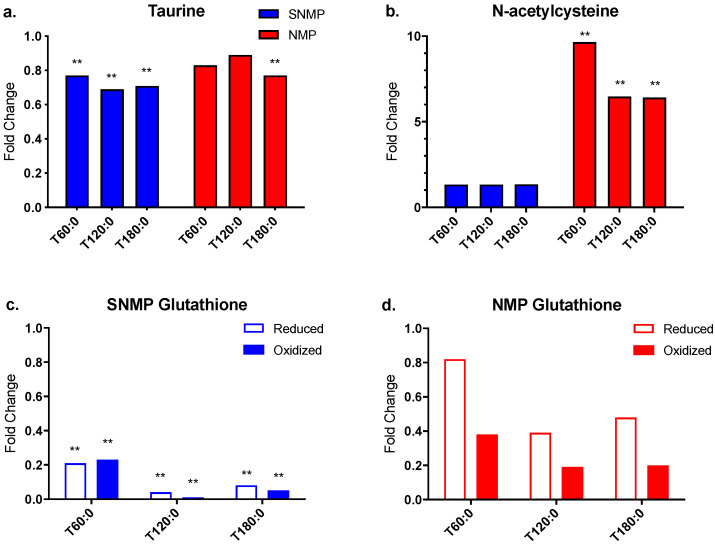
Oxidative stress metabolites during SNMP vs. NMP. (**a**) Taurine levels demonstrate a small but significant decrease during SNMP. (**b**) N-acetylcysteine levels are significantly increased during NMP. However, oxidized and reduced glutathione tissue concentrations are decreased in livers undergoing (**c**) SNMP and (**d**) NMP. Glutathione levels are significantly depleted during SNMP, but do not reach statistical significance during NMP. ** indicates *p* ≤ 0.05 and * indicates 0.05 < *p* < 0.10. Abbreviations: SNMP, subnormothermic machine perfusion; NMP, normothermic machine perfusion; x-axis represents fold change at 60, 120, and 180 min compared to pre-perfusion concentrations.

Four metabolites involved in antioxidant pathways were further examined: taurine, N-acetylcysteine (N-Ac), reduced glutathione (GSH), and oxidized glutathione (GSSG). Taurine tissue concentrations decreased significantly in SNMP livers (fold change range 0.69–0.77, *p* ≤ 0.05 for all) but only at 180 min in NMP livers (fold change 0.77, *p* ≤ 0.05) (Figure 7a). Tissue concentrations of N-Ac, an essential cysteine donor for glutathione synthesis [30], increased in both groups, although to a greater extent and with statistical significance in the NMP livers (fold change range 6.41–9.65, *p* ≤ 0.05 for all) compared to SNMP (fold change range 1.33–1.34, *p* not significant) (Figure 7b).

Glutathione is the most abundant thiol antioxidant in mammalian tissues and is composed of the amino acids cysteine, glutamic acid, and glycine [31]. Both GSH and GSSG tissue concentrations decreased significantly in SNMP livers indicating catabolic activity (GSH fold change range 0.04–0.21, *p* ≤ 0.05 for all; GSSG fold change range 0.01–0.23, *p* ≤ 0.05 for all) (Figure 7c). NMP livers also demonstrated decreasing tissue concentrations of GSH and GSSG, though to a lesser degree compared to the SNMP livers. In addition, tissue concentration comparisons to pre-perfusion levels in the NMP group did not reach significance (Figure 7d).

### 3.6. Bile Acid Metabolism in Livers during SNMP and NMP

Cholesterol and choline are essential substrates for bile acid synthesis. Cholesterol levels remained stable during SNMP but demonstrated a small, significant increase during the first 2 h of NMP. Choline levels increased significantly in both groups. With respect to primary bile acids, NMP livers showed a significant increase in taurocholate (fold change range 1.61–1.95, 0.05 < *p* < 0.10 at 60 min, *p* ≤ 0.05 at 120 and 180 min) and an increase in glycocholate sulfate, though without significance. SNMP livers demonstrated decreasing cholate levels that reached significance at 120 min and decreasing chenodeoxycholate levels that reached significance after 120 min (Supplementary Materials, Figure S2a–c).

## 4. Discussion

The study hypothesis was that physiologic changes taking place in the liver induced by normothermic versus subnormothermic perfusion modalities would identify metabolic processes, which could be leveraged to optimize future perfusion outcomes by mitigating IRI. We found that livers undergoing NMP demonstrated significantly greater tryptophan metabolism via the kynurenine pathway, which has significant clinical implications and therapeutic value. Livers undergoing SNMP showed a greater reduction in histamine, suggesting decreased inflammatory signaling, and impaired glutathione generation. In addition, SNMP livers demonstrated increased ATP and energy charge ratios compared to NMP livers. In light of these findings, several therapeutic strategies were identified for adjunct delivery during machine perfusion studies to optimize grafts for transplant.

One major finding of this study is the greater metabolism of tryptophan (TRP) via the kynurenine pathway (KP) in the NMP group with subsequently increased tissue concentrations of kynurenine (KYN) and kynurenate (KYNA). The difference in tryptophan metabolism during NMP compared to SNMP represents a clinically significant finding, as two studies found higher levels of KYN in transplanted livers that experienced primary nonfunction compared to livers with normal function [17,20]. Though KYNA levels were not reported, this highlights the therapeutic potential of this metabolic pathway. The activity of kynurenine aminotransferase (KAT), which converts KYN to KYNA [32], is temperature dependent and may explain why livers undergoing SNMP produced less KYNA. KAT exists as four isozymes (KAT I, II, III, and IV) in humans and rodents [32]. Both human and murine KAT II demonstrate maximum activity near 50 °C [33,34]. Decreased KAT activity may be a disadvantage of SNMP as KYN harbors pro-oxidant properties while KYNA has antioxidant properties [35]. Reassuringly, there was no significant accumulation of KYN during SNMP.

Greater tryptophan metabolism during NMP is further significant as the liver oversees 90% of TRP catabolism via the KP, which generates both pro- and anti-inflammatory signaling metabolites [36,37]. Two rate-limiting enzymes catalyze the conversion of TRP to N-formylkynurenine in the first step of the KP: tryptophan 2, 3-dioxygenase (TDO) and indolamine-2,3-dioxygenase (IDO). Although IDO is mainly expressed in extrahepatic tissues, it is also found in Kupffer cells, the resident macrophages in the liver involved in IRI [38]. Interferon-gamma (INF-γ) induced expression of IDO in Kupffer cells has been shown to promote immune tolerance via T cell apoptosis [39]. Similarly, greater TDO activity may also offer a potential immunosuppressive advantage as TRP catabolism by TDO has been suggested to play a role in immune tolerance of allogeneic liver transplantation by inhibiting T cell proliferation [40]. Therefore, one can consider enhancing TDO activity in SNMP livers to reap these immunosuppressive benefits. There are numerous studied positive regulators of TDO including glucocorticoids, TRP, and heme [36]. Upregulated TDO activity via glucocorticoid administration as part of routine post-transplant immunosuppressive therapy has been speculated to play a role in liver graft tolerance [40]. TRP supplementation, however, has been shown to negate TDO-mediated inhibition of T cell proliferation and IFN-γ production [40]. In this study, a synthetic hemoglobin oxygen carrier was used during NMP but not SNMP. As a result, a greater availability of heme may have contributed to the increased tryptophan metabolism seen in livers undergoing NMP.

Another potential enzyme target is kynurenine 3-monooxygenase (KMO), which catalyzes the conversion of KYN to 3-hydroxykynurenine (3-OHK), a pro-oxidant, neurotoxic metabolite that can induce endothelial cell apoptosis [35]. Compared to wild type, KMO knockout mice demonstrate decreased 3-OHK levels but increased KYN, KYNA, and anthranilic acid (AA) in the liver [41]. Furthermore, KMO inhibition has been shown to enhance KAT activity [42], which may be particularly beneficial during SNMP given lower functional activity of KAT at subnormothermic temperatures [34,35]. Shunting the metabolism of KYN from 3-OHK by KMO inhibition to KYNA may also be advantageous given the anti-inflammatory and immunosuppressive properties of KYNA [35]. Interestingly, there was no difference in NAD+ content between knockout and wild-type mice [41], suggesting NAD+ production is preserved in the setting of KMO inhibition.

With respect to histidine metabolism, livers undergoing SNMP demonstrated significantly lower tissue histamine concentrations than NMP livers, suggesting a potential reduction in the histamine-mediated inflammatory response during SNMP. Notably, a clinical study of transplanted livers using metabolomics found higher tissue histidine concentrations in grafts that experienced early allograft dysfunction compared to those with immediate function [18]. In the liver, histamine has been associated with biliary injury and hepatic fibrosis [43,44,45]. Porcine portal veins during hepatic IRI have demonstrated up to fourfold elevations in histamine [46]. In rat hepatocytes subjected to IRI, histamine reduced cell growth, enhanced oxidative stress, and promoted apoptosis [47]. Thus, livers undergoing NMP may benefit from histamine antagonism to prevent histamine release through degranulation. In the aforementioned study involving rat hepatocytes, the histamine H2 receptor antagonist cimetidine reduced the oxidative stress, apoptosis, and poor cell growth associated with histamine [47]. Cimetidine has also been shown to inhibit P450 activity, reducing subsequent endogenous production of reactive oxygen species [48]. Intrahepatic mast cells reside in the portal tracts and sinusoids of normal livers, though in low numbers [49]. Increased mast cell populations have been observed in liver pathologies, including fibrosis, nonalcoholic fatty liver disease, chronic hepatitis C, and hepatocellular carcinoma [50,51]. Doxantrazole and sodium cromoglycate are mast cell stabilizers with coincidental radical scavenging abilities [52], although their effects on hepatic IRI are not known. Investigating such H2 antagonists and mast cell stabilizers on histamine-associated inflammation in NMP livers may be a worthwhile future pursuit. 

Finally, the higher ATP and lower glutathione concentrations seen during SNMP mirror the findings from a similar study of machine perfused steatotic livers [21]. While the higher energy charge ratios are attributed to the lower metabolic activity at subnormothermic temperatures, the depletion of glutathione is likely due to the inability to expend ATP needed to resynthesize glutathione after its breakdown. Similarly, several clinical studies have demonstrated a correlation between higher concentrations of glutathione metabolites and early allograft dysfunction [17,18]. Therefore, supplementation of the circulating perfusate during SNMP with exogenous glutathione could be another potential therapy to improve a DCD graft’s ability to tolerate oxidative stress during transplantation.

There were several limitations to this study. First, sample sizes were small in the two perfusion groups. As this research relied on discarded human donor livers, there was limited availability of this precious resource and inherent variability in donor characteristics. To address the small sample size, multiple comparison corrections and false-discovery rates were taken into account for the untargeted metabolomic profiling. As a result, the analysis was able to demonstrate statistically and clinically significant differences between the two groups. In addition, the NMP group included one DBD liver whereas the SNMP group was comprised of only DCD livers. Future studies should examine DCD and DBD livers separately as they have been shown to exhibit different metabolomic profiles at the end of the static cold storage period [17]. To the best of our effort, donor demographics were otherwise similar. Livers with other skewing factors such as fibrosis or significant steatosis were excluded. Lastly, the decision to use a synthetic hemoglobin-based oxygen carrier instead of packed red blood cells (PRBC) was made in recognition of the possibility that PRBC may interfere with metabolic reactions and induce unwanted immunological responses. Compared to pooled PRBC gathered from several donors, HBOC-201 is also an immunologically inert oxygen carrier [53].

## 5. Conclusions

Non-steatotic livers undergoing normothermic versus subnormothermic machine perfusion differ broadly in their metabolomic profiles especially in terms of amino acid metabolism. Targeted enzyme inhibition and metabolite supplementation during machine perfusion offer potentially therapeutic methods for optimization of these metabolic pathways to mitigate IRI, allowing for the expansion of the donor pool to include livers that would have otherwise been deemed unsuitable for human transplantation.

## Figures and Tables

**Table 1:** Reasons for the discarding of donor livers.

Group	Liver #	Reason for Discard
NMP	1	No appropriate recipient, maximum cold ischemic time exceeded
	2	DCD with prolonged WIT
	3	DCD with prolonged WIT, history of alcohol abuse
SNMP	1	DCD with prolonged WIT
	2	DCD in donor >50 years of age
	3	DCD with prolonged WIT
	4	DCD with prolonged WIT

Abbreviations: NMP, normothermic machine perfusion; SNMP, subnormothermic machine perfusion; DCD, donation after circulatory death; WIT, warm ischemic time.

**Table 2:** Donor demographics of SNMP and NMP non-steatotic livers.

	NMP (*n* = 3)	SNMP (*n* = 4)	*p* Value
Age (years)	44 (28–60)	49 (33.5–52)	0.64
Gender (male)	2 (67%)	4 (100%)	0.43
BMI (kg/m^2^)	24.7 (16.9–32.5)	28.2 (26.1–32.1)	0.64
DCD Recovery	2 (67%)	4 (100%)	0.43
WIT (min)	34 (33–35)	30 (20–33)	0.14
CIT (min)	690 (360–930)	754 (685.5–861)	0.48
Liver weight (g)	1350 (1300–2200)	2139 (1646–2614)	0.28

Continuous variables presented as median with interquartile ranges. Abbreviations: NMP, normothermic machine perfusion; SNMP, subnormothermic machine perfusion; BMI, body mass index; DCD, donation after cardiac death; WIT, warm ischemic time; CIT, cold ischemic time.

## Supplementary Materials

The following are available online at https://www.mdpi.com/2077-0383/9/6/1864/s1, Table S1: Perfusate Compositions, Figure S1: NAD+ content in livers during SNMP vs. NMP, Figure S2: Bile acid composition during SNMP vs. NMP, Dataset S1: Complete untargeted metabolomic profile dataset.
