# Supplementary material for: Tryptophan Metabolism via the Kynurenine Pathway: Implications for Graft Optimization during Machine Perfusion
Source: J Clin Med. 2020 Jun 15;9(6):1864. doi: 10.3390/jcm9061864 (PMC7355886; doi:10.3390/jcm9061864)
Supplement: Supplementary file 1 [file jcm-09-01864-s001.zip › Supplementary materials v2.docx]

*Supplementary Materials*

**Tryptophan Metabolism via Kynurenine Pathway: Implications for Graft Optimization during Machine Perfusion**

**Anna Zhang ^1,2^, Cailah Carroll ^1,3^, Siavash Raigani ^1,3,4^, Negin Karimian ^1,3^, Viola Huang ^1,3^, Sonal Nagpal ^1,3^, Irene Beijert ^1,5^, Robert J. Porte ^5^, Martin Yarmush ^1,3,6^, Korkut Uygun ^1,3^, Heidi Yeh ^4,*^**

^1^ Center for Engineering in Medicine and Surgery, Massachusetts General Hospital, Harvard Medical School, Boston, MA 02114, USA

^2^ Tufts University School of Medicine, Boston, MA 02111, USA

^3^ Shriners Hospital for Children, Boston, MA 02114, USA

^4^ Division of Transplant Surgery, Massachusetts General Hospital, Harvard Medical School, Boston, MA 02114, USA

^5^ Division of Hepatobiliary Surgery and Liver Transplantation, University Medical Center Groningen, Groningen 9700, Netherlands

^6^ Department of Biomedical Engineering, Rutgers University, Piscataway, NJ 08854, USA

*Corresponding: Heidi Yeh, MD, Division of Transplant Surgery, Department of Surgery, Massachusetts General Hospital, Harvard Medical School, 55 Fruit Street, White 516, Boston, MA 02114, Telephone: +1-617-726-3664, Fax: +1-617-643-4579, Email: hyeh@mgh.harvard.edu

**SUPPLEMENTARY MATERIALS**

**Table S1.** Perfusate Compositions

| **SNMP Perfusate** | **NMP Perfusate** |
| --- | --- |
| Williams’ Medium E (2L)  Insulin (5 U/L)  Penicillin-streptomycin (40,000 U/L)  Hydrocortisone (10mg/L)  Sodium bicarbonate, 8.4% solution (as needed to titrate to pH 7.35-7.45) | Williams’ Medium E (1L)  Insulin (5 U/L)  Penicillin-streptomycin (40,000 U/L) Hydrocortisone (10mg/L)  Sodium bicarbonate, 8.4% solution (as needed to titrate to pH 7.35-7.45)  Fresh frozen plasma (1U or ~330 mL)  Human albumin, 25% solution (100 mL)  Heparin (5,000 U/L)  HBOC-201 (500 mL) |

Williams’ medium E (Millipore Sigma, St. Louis, MO, USA), Fresh frozen plasma (Research Blood Components, Boston, MA, USA), human albumin (CSL Behring, Kankakee, IL, USA), HBOC-201 (HbO2 Therapeutics LLC, Souderton, PA, USA), heparin (Sagent Pharmaceuticals, Schaumburg, IL, USA), hydrocortisone (Solu-Cortef, Pharmacia & Upjohn, Kalamazoo, MI, USA), insulin (Humulin R; Eli Lilly & Co, Indianapolis, IN, USA), pencillin-streptomycin (Invitrogen, Waltham, MA, USA). **Abbreviations**: **SNMP**, subnormothermic machine perfusion; **NMP**, normothermic machine perfusion.

**Figure S1. NAD+ content in livers during SNMP vs. NMP.** No significant change in tissue NAD+ concentration was seen in livers undergoing SNMP or NMP. **Abbreviations**: **SNMP**, subnormothermic machine perfusion; **NMP**, normothermic machine perfusion; x-axis represents fold change at 60, 120, and 180 minutes compared to pre-perfusion concentrations.

**Figure S2. Bile acid composition during SNMP vs. NMP.** (a) Cholesterol levels demonstrate a small significant increase in livers during initiation of NMP but do not change significantly during SNMP. (b) Choline tissue concentrations increase significantly in both groups at different time points. (c) Heatmap showing fold changes of primary and secondary bile acids (BA) during SNMP and NMP. ** indicates *p*≤0.05 and * indicates 0.05<*p*<0.10. **Abbreviations**: **BA**, bile acid; **SNMP**, subnormothermic machine perfusion; **NMP**, normothermic machine perfusion; x-axis represents fold change at 60, 120, and 180 minutes compared to pre-perfusion concentrations.
